# An Essay on the Use of Nitrate of Silver in the Cure of Inflammation

**Published:** 1829-04-01

**Authors:** 


### XIII.

An Essay on the Use of the Nitrate of Silver, in the Cure of Inflammation, Wounds, and Ulcers. By *John Higginbottom*, Nottingham, Member of the Royal College of Surgeons of London. Second Edition, much improved and enlarged. 8vo. pp. 204. 1829.

We think the work before us presents one of the most remarkable examples of the results of an intelligent and persevering investigation of an individual subject, which has ever fallen under our notice. It is now at least half a dozen years since the appearance of the first edition of Mr. Higginbottom's work. The multitude of interesting facts which are added in this, prove that the author's attention to the subject has been unremitting, and we think, must have conferred, in the course of their discovery, an ample reward of his labours.

In fact, the nitrate of silver has assumed, in the hands of our author, an importance both as to the efficacy and extent of its application in surgery, of which we did not think it susceptible. That to produce an eschar or vesication along the skin, by means of this remedy, would subdue inflammation, arrest erysipelas, preserve bruised parts,—frequently prevent suppurative—and constantly induce the adhesive inflammation,—are facts surely of the greatest possible interest. This remedy seems, indeed, under proper management, to possess an efficacy in modifying the morbid actions of parts of which it could not, a priori, be suspected.

It appears to us, from the perusal of this little volume, that the nitrate of silver applied along cutaneous and other surfaces, in a solid form, and in a prescribed manner and degree, induces a peculiar condition of the capillary vessels and circulation; and that, when these have been in a morbid state before, this morbid condition yields to that peculiar to the action of the nitrate of silver. The inflammatory, erysipelatous, and sloughing processes are thus changed to the adhesive, and if this remedy be applied, in the gentlest manner of course, even to a recent burn, the peculiarly painful affection which usually follows, is subdued and changed into that benigner condition peculiar to the action of this substance. How these effects are produced it is not easy to conjecture. Something appears to depend upon the union of the nitrate of silver with the animal substances of the part touched by it, by which an eschar is formed which must exclude the action of the atmospheric air. But it is also obvious that some more positive good is effected by the action of this remedy.

But we proceed to our more usual, and we think more useful and satisfactory, course of *analysis*.

Mr. Higginbottom's little volume is ushered in by a short introduction, in which he points out some advantages of the use of the nitrate of silver over the more usual modes of treatment, in navy, army, hospital, and domestic surgery. These advantages are chiefly its more commodious and portable form, its simpler application, and its prompter and surer efficacy in the cure of bruises or wounds and other injuries. These are indeed advantages; and we think it will well repay the trouble of those who are so situated as to undertake the task, to try the experiment.

In the first chapter we are informed that the application of the nitrate of silver is a cure for external inflammation. This point is further illustrated in chapter II, in which its use in phlegmonous inflammation, in whitlow, in erysipelas, and in inflammation of the absorbents is amply discussed, and illustrated by some truly interesting cases. The next point treated of is the efficacy of the nitrate of silver as a means of inducing the healing process, or adhesive inflammation. This subject is illustrated by cases and further observations in chapters III, and IV, in which the author treats of punctured and bruised wounds, at great length. In the third place we have the use of the nitrate of silver in healing ulcers; first, by inducing an adherent eschar, secondly, by the unadherent eschar, and lastly, in its conjunction with poultices. This subject is continued in chapters V, and VI, which treat of the cure of ulcers, and of old ulcers of the legs. The last, or seventh chapter, treats of the use of the nitrate of silver in burns and scalds. Two appendices are added, in the first of which the nitrate of silver is proposed as a vesicatory, and as a cure in gun-shot wounds, in neuralgia, in simple contraction of the rectum, in a peculiar ulcer of the tongue, &c.; and in the second, confirmations of the use of this remedy are given, from Mr. Webster, of Dulwich, and Mr. Tobias Browne, of Camberwell.

We think the following letter of Dr. Storer, particularly interesting. It is the unbiassed evidence of a looker on, who observes with accuracy, and infers with caution.

"Your late treatise had apprised me of your mode of using lunar caustic in the treatment of certain wounds and ulcers. I had not lost sight of the facts there detailed, and have been gratified to find, by repeated inquiries, that this plan of treatment continues to be confirmed by its success in your hands, and by other surgeons who have adopted it. It is now understood that it is capable of being extended with signal benefit, to old and extensively ulcerated legs; of which one case that I have seen is certainly an example. In cases of local disease, such success does not exceed the ordinary bounds of expectation; nor is it without analogy to the general application of this substance. When I heard that for some time past you had adopted the same plan of extinguishing cutaneous inflammation, not merely in symptomatic erysipelatous affection, but also in that constitutional erysipelas, which is often epidemic and always ushered in and accompanied with fever, I confess that I could expect no success from a practice militating so directly against the views generally entertained of the nature of this disease, and would give no ear to it without ocular demonstration of its utility.

"I have now seen two cases of constitutional erysipelas treated upon this principle, and as far as two cases well marked will go to authorise a conclusion, mine must be greatly in favour of the practice.

"The first was that of a healthy young woman, upwards of twenty years of age, seized with rigor, followed by smart fever, and on the second day by a complete erysipelas of the face, extending to the hair on the forehead. The fever continuing with a further extension of the inflammation, after bleeding, purgative and saline medicines, and a small vesication having arisen on the cheek, it was judged necessary on the third day, to apply the caustic over the whole extent of the erysipelas. The patient's own account was, that she suffered considerable pain for ten hours: but from that time, the feverish symptoms ceased, and the inflammation was arrested and subdued.

"When I saw her the fourth day after the application of the caustic, there was neither fever, nor any remains of erysipelas. The face was as black as that of an African, but in a few days more, I found her free from all symptoms of disorder, the epidermis peeling off, and the complexion underneath quite natural.

"The other was a fine healthy boy of sixteen months old, who after a feverish attack had erysipelas on one hip and thigh, and extended partially to the leg. In this case also the fever and inflammation had subsided in forty eight hours and upwards, after the application of the caustic. The account of the child's mother was that it cried very much for one hour after, then fell into a long and calm sleep, out of which it waked without fever, and calling out for food. In three days afterwards when I saw the child, it was quite well, and the scarf skin of the inflamed parts separating.

"This is the extent of what I have observed of this novel mode of treating erysipelas, and which has surprized as it has satisfied me. Sincerely wishing success to your endeavours to improve the practical part of your profession,

"I am, &c. JOHN STORER." 42.

To this letter we may subjoin one from Dr. Marshall Hall, on the efficacy of the nitrate of silver in subduing inflammation of the absorbents.

"I have had an interesting opportunity of watching the influence of the nitrate of silver in a case of inflammation of the absorbents, and I will endeavour briefly to state the obvious effects of this remedy. The case was that of a young lady, aged twelve years; it began by a chilblain upon the heel; inflammation of the absorbents up to the groin followed; suppuration took place in twenty-four spots. The nitrate of silver was not applied during the first five weeks; this was very much regretted after its effects had been observed. The patient was seen by Mr. Lawrence and Mr. Wardrop, who both expressed themselves much interested in it.

"As the case would be long, I shall simply enumerate the effects of the application of the nitrate of silver in this case, as they presented themselves to my observation:—

"1. It prevented suppuration in many places where the redness and tenderness were recent, yet such as before had inevitably led to the formation of pus.

"2. If suppuration had taken place, the tenderness was still promptly removed and the pus from being thick, white, and opaque, was rendered first thin and somewhat limpid, and perhaps streaked with blood, and then by degrees perfectly watery and limpid.

"3. When the pus approached the surface, a small opening formed, by which it exuded, and it became unnecessary to use the lancet.

"4. The abscess was far more disposed to heal than any of those to which the nitrate of silver had not been applied.

"I may add that in several other cases I have seen the application of the nitrate of silver early, along the course of the inflamed absorbents, subdue the disease at once, rendering all other remedies totally unnecessary. I believe this would have been the case in the instance mentioned, had this remedy been applied very early.

"I am, &c. MARSHALL HALL.

"P. S. It may be well to subjoin the outline of another case, in which the nitrate of silver was useful.

"A little girl fell and cut the ring-finger. The joint became permanently inflamed and swollen. The affection had subsisted three months when I saw it, preventing the little patient from attempting the piano-forte. The nitrate of silver was applied every fourth or fifth day, so as to form an eschar over the whole inflamed surface, but not so as to give pain. The finger was quite well in a few weeks.

"The powers of this remedy in subduing external inflammations are altogether very remarkable, and, I think, not at all generally known." 57.

The objects in the application of the nitrate of silver in punctured wounds, are to secure a free exit for pus, and to subdue inflammation. We have only space for the following cases.

"*Case* 18.—A servant, aged twenty, slightly wounded the fore part of the index finger, at the first joint, by a bone of a hare, in dressing it. The wound healed in a day or two, and no notice was taken of it. A few days afterwards the finger became swelled and painful, and affected with diffused inflammation of an erysipelatous character, extending to the back of the hand, and there it was bordered by a ring of a more vivid colour. I applied the nitrate of silver nearly all over the finger, and upon the back of the hand, upon and beyond the inflamed border.

"On the following day the swelling remained as before, and the patient complained of heat of the parts to which the application had been made, and a small part of the finger which had not been touched was very painful. I applied the nitrate of silver to this point.

"On the succeeding day, the swelling had become puffy, and the whole finger and hand were free from pain. A few days afterwards, the hand was quite well. No medicine was given, nor was the patient prevented from pursuing her usual avocations.

"This case was the more interesting because I had two similar ones a short time before, which had been occasioned by wounds received in cutting dogs' meat, and which, under the ordinary treatment, had been several weeks in getting well." 69.

"*Case* 25.—This case illustrates the mode of treatment by the nitrate of silver, of some of those terrible effects of punctured wounds, which have been neglected in the beginning.

"B. Unwin, aged forty, washerwoman, applied to me on July 10, 1820, with severe inflammation and ulceration of the middle finger, arising from a puncture by a pin or needle, some time before. There was much painful tumefaction, and the integuments had burst along nearly half the length of the finger, on the ulnar side, and over the middle joint, on the radial side. The probe did not, however, pass to the bone, or into the joint. I applied the nitrate of silver deeply in every part, and over the whole surface, and enveloped the finger in a cold poultice covered with cold water.

"On the eleventh, she reported that she had slept well for the first time for a whole fortnight. There was scarcely any pain, but she complained of soreness; the swelling had greatly subsided. The nitrate of silver was again applied, and the poultice and lotion continued.

"On the twelfth there were still swelling and pain; there was considerable bleeding from the wound, so that I did not apply the caustic well.

"On the thirteenth the swelling and pain were nearly gone. I repeated the application of the nitrate of silver, which induced bleeding from a fungous growth.

"On the fourteenth the swelling had nearly subsided, and the cuticle was separated all over the finger. The nitrate of silver was applied extensively over the wound, and abraded parts. It induced little bleeding, or pain.

"On the fifteenth the fungus was nearly removed; the wounds presented an appearance of slough over their surface. The nitrate of silver was applied to the fungus, which still remained.

"On the seventeenth the wound was much smaller, and the slough was separating. The nitrate of silver, and cataplasm were applied as before.—A similar report was made on the succeeding day.

"On the twentieth the slough was separating. The nitrate of silver and cataplasm were applied.—A similar report was made on the twenty-second.

"On the twenty-fourth, the slough having separated, the integuments over it were found to be flabby and loose.—The nitrate of silver was applied to them.

"By a continuation of this plan the wound gradually contracted, and at length, when there was no further use for the cataplasm, the eschar became adherent, and the ulcer healed underneath. It appeared highly probable to me, that under any ordinary treatment, the finger in this case, would have been lost." 80.

The chapter from which these cases are extracted, treats of simple, inflamed, neglected, and large punctured wounds,—of wounds received in dissection,—and of the bites of animals. It consists of a detail of cases in exemplification of the efficacy of the treatment by the nitrate of silver.

The succeeding chapter treats of simple, inflamed, and severe bruised wounds, and of bruised wounds with slough. The following is an example of the last of these.

"*Case* 47.—As two men were carrying a heavy bundle of iron rods, it slipped from the hands of one, and fell on the toe of the other. The blow was so severe, as to cut through a strong shoe, and to shatter the nail of the great toe, and drive it into the surrounding integuments, and to cause a slight fracture of the bone of the first phalanx of the great toe, which was exposed. The case was so severe that I almost resolved to remove the toe; but finding the integuments underneath in so bruised a state, that I could not depend upon them as a covering, I wished to try my new practice, with the nitrate of silver. After removing the portions of loose nail, I applied this remedy over the whole skin of the toe, and on as much of the wound as was exposed after the parts were closed by the hand of an assistant. I then applied the adhesive plaster, so as to retain the parts together; and over it two or three folds of linen, moistened with cold water.

"The patient complained of some pain for three days, and then became easy. I did not examine the wound for nine days. I then found it in a quiet state, and free from inflammation; the surface of the sore appeared to be covered with a layer of coagulable lymph. I again applied the nitrate of silver, and over the eschar, first lint, then a plaster of the neutral ointment, and then adhesive plaster.

"In other three days the sores were nearly healed, except the part occupied by the nail, which had been removed at my first visit.

"I applied the nitrate of silver every third or fourth day, for four times, when the wound was quite well. A new nail was making its appearance about the end of three weeks after the accident." 109.

The fifth and sixth chapters treat of the healing of ulcers. The treatment by the nitrate of silver is so successful, yet the directions required to be followed so minute, that we must refer to the work itself for them. We think it will amply repay those who have such cases to treat, to observe Mr. Higginbottom's directions; indeed, we do not conceive it would be right to do otherwise. We insert one case only illustrative of this subject.

"*Case* 61.—M. Bosworth, aged sixty-nine, has had ulcerated legs for the last thirty-five years, and, during that interval, has had the ulcers healed several times, but for short periods only. The present ulcers on both legs are about twenty in number; two are of the size of a crown piece; the others from the size of sixpence to that of a pea; the veins are in a varicose state, and the legs are extensively inflamed; his sufferings have been very great; his nights have been entirely restless from excessive heat and pain. He has been scarcely able to follow his employment, of a framesmith, for many years, and he has almost always been obliged to sit to his work. I applied the nitrate of silver on the whole ulcerated and inflamed surfaces, after he had rested in bed, with poultices on the ulcers, for twenty-four hours; and I gave him a blue pill and purging powder.

"At my next visit he said that he had not suffered more pain from the application of the nitrate of silver, than from some of his former dressings; that, on the following day, he had experienced scarcely any pain, and that he had had better nights than for many years.

"I examined the sores on the fourth day, and found them in a healing state; the inflammation was gone, and my patient suffered no pain. The nitrate of silver was reapplied, with the addition of Mr. Scott's mode of bandaging.

"This case was dressed every third or fourth day, for ten weeks, when all the ulcers were perfectly healed. During the whole of this time he followed his employment with more ease than he had done at any former period for thirty years.

"I directed the patient to continue to come to me twice a week, for a few weeks, that I might examine the bandages and apply the nitrate of silver to any tender or slightly inflamed spot, if any such should make their appearance." 142.

The last chapter upon burns and scalds is not the least interesting; but we must pass it over very briefly, extracting only one short paragraph.

"I have found that, by slightly passing the nitrate of silver once over a burnt surface, the pain is increased for a short time, but then totally subsides, vesication appearing to be prevented; the black cuticle peels of in a few days, leaving the part well. In cases in which the cuticle has been removed, the nitrate of silver applied on the surface, induces an adherent eschar, and prevents the consequent ulceration. In cases in which a slough covers the surface, I have removed it with the scissors and forceps, and applied the nitrate of silver, and have cured them by the unadherent eschar.

"In one case, in which, after a burn, the part was healed over, and a considerable cicatrix formed resembling a fungus, and attended with severe pain, the nitrate of silver, applied as in external inflammation, removed all inflammation and pain." 150.

From Mr. Webster's letter, in the appendix, we take the following interesting observations on the use of the nitrate of silver as a blister.

"I understand that Mr. Higginbottom has thought of using the nitrate of silver as an Epispastic. Having observed the vesicating properties of this remedy, when applied merely to produce blackness of the skin, I was induced to apply it as a blister, and from the numerous trials I have given it with this view, I have no doubt that were it generally applied by the profession it would soon supersede the use of cantharides; of warm water, as proposed by some; and of the heated metallic plates of Sir Anthony Carlisle.

"The advantages of the nitrate of silver over cantharides, the usual epispastic, are five-fold.

"1. It acts much more *quickly*, requiring generally only from one to three or four hours.

"2. It produces very much *less pain* in its action, independently of lessening the *duration* of that pain.

"3. It causes scarcely any constitutional irritation, even in children.

"4. It does not affect the bladder, nor bring on strangury.

"5. It heals much sooner.

"The mode of application is simply to wet or rather *moisten* the space to be blistered, with water, and then to rub a stick of the nitrate three or four times over the part, according as the cuticle is thin or otherwise. Once or twice is sufficient to blister the skin of children, or where the cuticle is thin and loose. In a few minutes the part begins to smart a little, which generally does not last long, and in the course of from two to four or five hours the blister insensibly rises. The serum is to be discharged and the part dressed, as in a common blister, but as it heals sooner, any application to keep up a discharge, must be applied at the first dressing, and for this purpose the cuticle must be entirely removed when the serum is discharged.

"It may perhaps be objected by some to this new mode of blistering, that there is a dread of the nitrate of silver producing a deep eschar—but this is not the case, the cutis vera is not affected in this way, at least when the nitrate is only applied to the cuticle, nor do I believe that nitrate of silver ever produced a *deep eschar* under any circumstance, however freely applied.

"Whether the nitrate of silver produces any specific or peculiar effect on internal extensive inflammations I am not prepared to state, but I believe that it is capable of producing all the beneficial effects of the common blistering plaster, without its troublesome ones." 199.

We have now but to sum up our remarks in a short character of this little work. We would say, then, that Mr. Higginbottom has done a most essential service to the profession by thus tracing out the virtues of the nitrate of silver, and so adding, as it were, a useful remedy for inflammation, wounds, and ulcers, to those which we possessed before; for, in these points of view, we regard this little work as perfectly original. The author has, moreover, proved himself capable of pursuing a new train of ideas—a new course of investigation; and we think, with him, that more useful facts will still flow from further inquiry into the effects of this remedy, especially in regard to some internal diseases. We cordially recommend Mr. Higginbottom's labours to the consideration of our readers.

